# A qualitative study of community home-based care and antiretroviral adherence in Swaziland

**DOI:** 10.7448/IAS.16.1.17978

**Published:** 2013-10-08

**Authors:** Robin Root, Alan Whiteside

**Affiliations:** 1 Department of Sociology and Anthropology, Baruch College, City University of New York, One Bernard Baruch Way, New York, NY, USA; 2 Health Economics and Research Division (HEARD), University of KwaZulu-Natal, Durban, South Africa; 3 CIGI Chair in Global Health Policy, Balsillie School of International Affairs and Wilfrid Laurier University, Waterloo, Canada

**Keywords:** Africa, HIV, AIDS, antiretroviral treatment, ART, adherence, stigma, home-based care, chronic disease

## Abstract

**Introduction:**

Antiretroviral therapy (ART) has rendered HIV and AIDS a chronic condition for individuals in many parts of the world. Adherence, however, is integral to achieving chronicity. Studies have shown both relatively high ART adherence rates in sub-Saharan Africa and the importance of community home-based care (CHBC) to facilitating this process. In light of diminished HIV and AIDS funding globally and increased reliance on CHBC throughout Africa, a better understanding of how CHBC may strengthen ART adherence is essential to improving patients’ quality of life, tending to the needs of care supporters and achieving healthier populations.

**Methods:**

This article reports findings from a qualitative study of a CHBC organiztion serving an estimated 2500 clients in rural Swaziland. Semi-structured questionnaires with 79 HIV-positive clients [people living with HIV and AIDS (PLWHA)] yielded data on diverse aspects of being HIV positive, including insights on whether and how PLWHA perceived care supporters to facilitate ART adherence in a high stigma and structurally impoverished setting.

**Results:**

Ninety-two percent of participants said their health had improved since care supporters came into their lives. A major finding was that an estimated 53% of participants said they would have died, a few from suicide had the care supporter never intervened. More than one in four participants (27.9%) sought HIV testing after a care supporter began visiting them. Nearly a third (31%) commenced ART after and largely as a consequence of care supporter intervention. Approximately one in four (23%) reported that their care supporter had helped them to disclose their HIV-positive status to family members. Twenty-seven percent said they had felt discouraged or had been discouraged from taking ART by members of their family or community.

**Discussion:**

General inductive analysis of participant reports suggested two social mechanisms of CHBC impact on ART adherence: (i) cultivating client-care supporter “talk” to enhance treatment uptake and literacy, reduce felt stigma and challenge social pressures to desist from ART and (ii) real-time interactions between clients and care supporters whereby the care “relationship” was itself the “intervention,” providing lay counsel, material and financial assistance, and encouragement when clients suffered stigma, side effects and other obstacles to adherence. These social dynamics of adherence generally fall outside the purview of conventional clinical and public health research.

**Conclusions:**

PLWHA reports of care supporter practices that enabled ART adherence demonstrated the pivotal role that CHBC plays in many PLWHA lives, especially in hard to reach areas. Relative to clinic personnel, care supporters are often intensely engaged in clients’ experiences of sickness, stigma and poverty, rendering them influential in individuals’ decision-making. This influence must be matched with on-going training and support of care supporters, as well as a clear articulation with the formal and informal health sectors, to ensure that PLWHA are correctly counselled and care supporters themselves supported. Overall, findings showed that PLWHA experiences of CHBC should be captured and incorporated into any programme aimed at successfully implementing the Joint United Nations Programme on HIV and AIDS (UNAIDS) Treatment 2.0 agenda Pillar 4 (increasing HIV testing uptake and care linkages) and Pillar 5 (strengthening community mobilization).

## Introduction

In many parts of the world, community home-based care (CHBC) has long played critical roles in HIV and AIDS policy and programming [1]. More recently, expanded rollout of affordable antiretroviral treatment (ART) has transformed HIV and AIDS into a chronic condition for many HIV-positive individuals – people living with HIV and AIDS (PLWHA) [2, 3]. Twinning these agendas [4–6], properly designed CHBC has been shown to strengthen ART adherence in resource-limited settings [7]. To date, CHBC-focused studies have provided important insights regarding the frequency and severity of clients’ symptoms [8], the benefits of home-based HIV and TB testing and counselling [9], family and household stressors [10], caregiver health status [11] and integration of CHBC into care continuums [6]. However, little research has explored the “changing nature of care” heralded by ART [12, p. 2]. To illuminate the “changing nature of care” and CHBC experiences in rural Swaziland, this article utilizes a qualitative health research framework to examine PLWHA perceptions of care supporter practices that they believed helped shape ART adherence.

Adherence, as the framing concern of this article, is conceptualized as a biomedical/public health construct within which are housed socially mediated experiences of being HIV positive. Identifying the structural and sociocultural dynamics of successful adherence from PLWHA perspectives is essential for designing effective interventions. Furthermore, a deeper understanding of ART adherence in sub-Saharan Africa (SSA) would help to shed light on why adherence in SSA is reportedly higher than in North America [13]. PLWHA reports of CHBC practices are especially insightful because they detail the extensive burdens care supporters shoulder as task-shifting devolves health services from medical to community-based workers [14, p. 35–36]. The findings reported here are timely and important because the government's National Strategic Framework for HIV and AIDS (2009–2014) has identified CHBC as a core component of its plan. Findings foreground the need for substantial rather than symbolic support of individuals and organizations providing home-based care, including healthcare, material assistance and continuous training for care supporters [15]. Given the integral and expanding roles of CHBC, findings are relevant to many settings in rural Africa where affordable ART is available but access to formal health services is lacking and entrenched stigma undermines individuals’ attempts to develop HIV healthy practices. The authors conclude with recommendations to optimize the critical human and material resources that effective and sustainable CHBC requires [16].

## Background

A British protectorate until 1968, the Kingdom of Swaziland is a landlocked nation bordered by South Africa and Mozambique. The smallest country in Africa, it is perhaps best known as the last absolute monarchy on the continent. Economically, despite its lower middle-income ranking [17], a majority of the population is unemployed (70%), living in chronic poverty (69.2%) and unable to meet basic food needs (66%) [18]. HIV prevalence in adults is among the highest in the world (26%) where infection rates peak among women (49%) aged 25 to 29 years and among men (45%) aged 35 to 39 years [19]. Some scholars have argued that this decade difference in peak infection rates implicates age-disparate or intergenerational sex between older men and younger women as an indication of broader gender vulnerabilities [20, 21]. High HIV and AIDS-related morbidity and mortality among young adults has pushed the rate of orphaned and vulnerable children to 45% and increased the numbers of child-headed households [22]. Between 2000 and 2009, life expectancy fell precipitously from 61 to 32 years [23] but recently has turned upward to nearly 49 years [24]. Concomitant with HIV and AIDS, the population of 1.1 million suffers the highest tuberculosis (TB) rate in the world [25, 26, p. 5].

To meet these challenges, the government aims to integrate and decentralize health services [27, 28, p. 60]. CHBC figures importantly in its plans to do so. By 2014, the National Strategic Framework states that 80% of households receiving CHBC should report the care to be “relevant, comprehensive and of good quality” and, second, that 50% of PLWHA with advanced HIV infection should receive palliative care from CHBC [29, p. 57]. Given these substantial demands on community resources, in-depth qualitative research is well-suited to generating insights on the most valuable CHBC resource: people.

## Regional health resources and collaborating organization

A 2006–2007 mapping of health service availability reported dire shortages in health resources throughout the country [30]. With 10 physicians per 100,000 inhabitants and only 11 social workers and 2 psychologists nationwide, there is insufficient capacity in the formal sector to meet PLWHA's clinical and psychosocial needs. Of the country's four regions, Shiselweni, where research for this study was conducted, is the most underserved. According to the government mapping, doctors in Shiselweni number 15 per 100,000 inhabitants and community health workers 174 per 100,000, both of which are inadequate to the area's healthcare needs. Only 11% of health facilities in Shiselweni were staffed with a health worker trained comprehensively in HIV and AIDS. No health facility at the time of the mapping had Internet access.

This is the healthcare setting in which the Shiselweni Home-Based Care Organisation (SHBC), a registered non-governmental organization in Swaziland and South Africa, endeavoured to provide a range of home-based HIV prevention and treatment support services. Between 2006 and 2011, the SHBC expanded rapidly from 32 to 750 volunteer care supporters to serve 2500 clients in 27 communities across approximately 2200 km^2^. Care supporter training takes place over one week and is provided by two care coordinators who have been educated in the basic knowledge of HIV and AIDS primary prevention, HIV testing, confidentiality protection, antiretroviral treatment, treatment adherence and positive (secondary) prevention. Training also includes a religious component, which the founder deemed relevant, in part because of the high percentage (86%) of Swazis who identify as Christian (86%). Part of the SHBC's Christian ethos “To Become the Hands and Feet of Christ” is to provide care without monetary remuneration. Though concerns have been raised in the home-based care literature that lack of compensation taxes an already overburdened and under-resourced cohort comprised mostly of women [31], participants described the unique capacity of a “Christian heart” to provide exceptional care. Logistically, care supporters travel in pairs and record each home visit. These records culminate in monthly reports detailing, per community, the number and gender of care supporters; the number and gender of clients; total number of home visits; number of new clients; and of those who have moved, died, are terminal and/or who have chronic ailments. From 2008 to 2011, a total of 7893 people were referred for ART; 1001 clients were on ART; and the mortality rate of SHBC clients declined from 35.3 to 14.8% [32].

## Methods

The findings reported here are derived from a larger qualitative study of the sociocultural aspects of AIDS in Swaziland [33–35]. Because qualitative methods have been shown to be effective in investigating health in developing countries [36], a descriptive qualitative research design was deemed appropriate to a focused study of PLWHA experiences of CHBC in a rural resource-limited setting. The main concept – PLWHA experiences of CHBC – was operationalized in terms of the following variables: clients’ critical needs; social networks for meeting those needs; perceptions of SHBC care supporter practices; HIV and AIDS communication with community care supporters; family reactions to the care supporter; personal religiosity and the significance of care supporters’ Christian affiliation. These variables in turn became the basis for designing the semi-structured questionnaire, which included 36 structured and 15 open-ended questions. The variables and questions were derived from the first author's previous and on-going investigation of social aspects of HIV and AIDS in Swaziland [33–35].

Oral semi-structured, face-to-face questionnaires were conducted with 79 individuals from 11 of the total 27 communities served by the case organization (Table 1). Purposive sampling criteria were that the individual had been diagnosed with HIV and was a current SHBC client. Participants were notified of the study approximately one month in advance of data collection by their care supporters and invited to participate on a volunteer basis. Oral informed consent was secured in person before the questionnaire was conducted. Key informant interviews provided important contextual insights. These included the organization's regional coordinator, who manages approximately 750 care supporters, and the group's nurse trainer. The research protocol was reviewed and approved by Institutional Review Board of Baruch College, the City University of New York.

**Table 1:** Study participants – key demographics

Sex	% (*N*)
Male	35.4 (28)
Female	64.6 (51)
Total	100 (79)
Age	Years
Average	44.4
Range	17–75
Age range distribution	% (*N*)
17–24	5.1 (4)
25–35	20.3 (16)
36–44	30.4 (24)
45–54	21.5 (17)
55–75	22.8 (18)
Total	100.1 (79)
Schooling	% (*N*)
No schooling	21.5 (17)
Primary	54.4 (43)
Secondary	17.7 (14)
High school	5.1 (4)
Others (Sebenta, adult education)	1.3 (1)
Total	100 (79)
Marital status	% (*N*)
Single, unmarried (includes some cohabitation)	44 (35)
Married (Christian and/or Swazi custom)	22 (18)
Widowed	30 (24)
Divorced/separated	3 (2)
Total	100 (79)

The semi-structured questionnaire was translated into siSwati by a leader in the case organization and then back-translated for confirmation. With the assistance of interpreters from the case organization, the majority of face-to-face questionnaires were audio recorded and conducted by one of the study's principal investigators (*N*=48) and a Fulbright Scholar (*N*=27). Responses were summarized in writing during questionnaire administration. The questionnaires took on average approximately 30 minutes per participant and were conducted in diverse community settings: inside churches, when services were not being held; in churchyards, outdoors; and at a chief's residence (*umphakatsi*), an orphan care point (*gogo center*) and a chief administrative center (*inkhundla*). One participant, too unwell to travel, was interviewed in his home.

Concurrency of data collection and analysis, where data collection and analysis function iteratively to produce new knowledge, is characteristic of much qualitative research [37]. Thus, when it was evident during data collection that a question was eliciting redundant responses, or not eliciting noteworthy responses, it was discontinued. By the same token, if early reports indicated an experience worth tracking across the remainder of the cohort, questions were added. Allowing for a more focused and nuanced probe of participants’ experiences than strictly structured surveys, these on-site iterative adjustments to the instrument resulted in a different denominator for some responses.

Structured and semi-structured responses were subsequently entered into Qualtrics survey software for analysis. The distinction between qualitative and quantitative research is often misleadingly construed as discretely dichotomous, when in fact “research across the social sciences relies on a balanced, common sensical mix of both kinds of data” [38, p. 2]. Qualitative data, especially in health-related research, are often non-numeric content that can be calculated for frequency and interpreted for meaning, thus appropriate to both quantitative and qualitative analyses [39]. In this study, structured responses were calculated to generate descriptive statistics that helped to identify patterns, as well as outliers, in participant experiences. Such responses included, for example, rates at which care supporters facilitated ART uptake. Semi-structured (open-ended) responses included, for example, descriptions of what participants felt would happen were the care supporter no longer able to visit them. General inductive analysis of relevant data generated deeper thematic categories that helped to organize and articulate participants’ CHBC experiences of ART adherence support.

## Study limitations

Purposive sampling for participant recruitment limits the generalizability of study findings. Also, because participants were largely self-selected, findings may disproportionately shed light on clients who were in better health and who therefore may have experienced better CHBC practices than clients who did not participate in the study. Approximately twice as many women as men were represented in the sample. Without a research framework explicitly designed to explore gendered differences, however, it is not possible to extrapolate the significance of this differential participation. Use of an interpreter from the case organization may have constituted a limitation as well as an advantage. The impact was indeterminate since that person may have shaped an atmosphere of openness or potential concealment. Because SHBC care supporters enjoy reputations as guardians of confidentiality, their involvement likely was not limiting in this regard. Also, in paraphrasing participant responses, interpreters may have influenced content in ways there were difficult to ascertain. Finally, ART adherence was self-reported. Since the aim of this article is to describe the subjective challenges of adherence, actual adherence levels did not detract from interpreting study findings regarding what PLWHA felt helped them to achieve adherence.

## Results

### Study sample

Approximately two-thirds (64.6%) of the study sample were women and the remainder (35.4%) men (Table 1). Participants’ ages ranged from 17 to 75 years, averaging approximately 44 years. About half (50.7%) were aged 25 to 44 years. Nearly one in four participants (22.8%) were aged 55 or older. Nearly 18% had attended secondary school and 5% high school. The educational attainment of more than half (54.4%) was limited to some primary schooling. A sizeable proportion (21.5%) of the sample reported that they had received no schooling. Forty-four percent of respondents reported that they were single/never married, though many cohabitated with a sexual partner. Approximately one in five indicated that they were married, and nearly a third said that they were widowed.

It is important to note that conventional demographic categories such as “marital status” mask the complex marital, familial and sexual realities beneath these rubrics that may impact individuals’ experiences of HIV and AIDS and thus care supporters’ strategies for supporting adherence. An example of this demographic dissonance was a male participant who had had three wives. One had died and the other two divorced him. Because one wife had died, he was categorized as widowed, though such labelling eclipses the social fact that two wives had left him. To label him “divorced” would have overlooked that a spouse had died. Such “lived realities” intimate some of the socio-cultural, economic and sexual dynamics that participants and their care supporters often navigated in pursuit of survival and a state of wellbeing.

### Elevating health: HIV testing, disclosure, ART practices

An overwhelming majority (92%) of participants felt that their health had improved since a care supporter began visiting them at home. More than one in four participants (27.9%) sought HIV testing after a care supporter began visiting them. Combined with reassurance and encouragement that proactive steps could be taken if they tested HIV positive, a number of clients felt that without the gentle push to test, they might not be alive. Nearly one in three participants (31%) commenced ART after and largely as a consequence of care supporter actions to educate, encourage and assist them in obtaining testing and treatment.

Participants described the synergies from care supporters’ advice when it echoed and supplemented counsel that they had been given at the clinic. “Sometimes there are side effects,” said a 60-year-old man, “and the care supporter is always there for me, telling me, ‘go straight to the doctor and tell him’.” Medication regimens explained to patients at the clinic required translation into individuals’ daily practices. This translation was more likely to be sustained if a person such as a care supporter was willing and able to play this role. Care supporters emerged as especially pivotal when clients experienced apparent medication side effects by encouraging clinic follow up and counselling food intake. On-going instruction regarding ART adherence (“they helped me to take the pills: eat first, wait, then take pills – I used to vomit”) and encouragement (“life continues even when one is HIV positive”) were cited as key factors in why a care supporter relationship was felt to be effective.

A large majority (86%) reported that they viewed care supporters as both “health” and “religious” persons’ [40]. A complete analysis of the role of religion in participants' experiences of CHBC was beyond the scope of this paper; however, preliminary results have been reported [40]. Though approximately 15% of participants indicated that they did not identify as Christian, nearly every participant felt it was important that a care supporter be a Christian, in part because Christian care supporters were felt to have a larger “heart” than a non-Christian care supporter, to be trust-worthy and better able to maintain confidentiality. Asked whether there were religious aspects to the home-based care they received, most participants reported that there was; this included primarily prayer and Bible reading. The actual tasks care supporters performed were felt to reflect and constitute an ideal Christian. “She cares for me,” said one participant. “She wants to know, did I get food. She reminds me of the days of going to the hospital to get some ARVs, if it is my date.” The religious aspects of care support sometimes affected HIV-related behaviours, such as adherence. “When they [the care supporters] come, they pray and encourage me to believe in God,” said one participant. “[And they counsel] that whenever I take ARV, I must also pray to God because He is the one who cares [about] our lives.” Religion and adherence were integrated into the home-based care experience of another participant, who said the care supporters “greet me, pray, preach and say continue ARVs.”

Participant responses to the question of would happen if their care supporter could no longer visit them intimated both the clinical and psychosocial dynamics of care supporter impact on self-efficacy and ART adherence: “Life could still go on,” said a 62-year-old man who had been concerned when the care supporters first arrived that they would gossip, “because now I've got the knowledge [of how to live] from the care supporter and the clinic.” Their encouragement to adhere, he said, was integral to his being able to hold the hoe when cultivating and to feeling strong enough to weed. Similarly, a 30-year-old man explained: “The caregivers teach me how to care for myself with this condition [HIV] and to live my life.”

Care supporters’ agility at creating safe disclosure settings was felt to be one of their most important practices. Safe disclosure is often essential to strong adherence, since not disclosing and fear of stigma are among “the most important and prevalent factors … to negatively affect adherence in sub-Saharan Africa” [13, p. 687]. In this study, approximately one in four (23%) participants reported that their SHBC care supporter had helped them to disclose to family members. Disclosure-brokering practices included discussing strategies and, at times, joining the participant in disclosing to the family. Having counselled that at least one family member should be cognizant of the client's HIV status, a care supporter might also, with the client's permission, inform a family member on behalf of the client. Importantly, in light of the reported significance between perceived stigma and missed ARV doses in Swaziland and elsewhere [41] disclosure could feel unproductive and, at times, be detrimental to PLWHA wellbeing. A male participant in this study said he had disclosed his HIV-positive status to his family before a care supporter had entered his life. His family's response, recalled the man, who estimated his age as over 60 years, was to laugh at him and call him stupid for revealing such confidential information. A care supporter's aim, thus, was to create the household conditions for productive disclosure: an HIV disclosure that enabled HIV healthy practices. A 39-year-old woman described how her care supporter had counselled her to tell her five children (aged 7 to 26 years) of her HIV diagnosis. The woman felt her “mind was not in good condition” and hoped her children would remind her of when to take her medications. This was especially important to the participant since the children had seen their father, who reportedly had refused to take ARVs, die.

### ART discouragement: three modalities

Exacerbating the structural challenges individuals faced in their struggle to achieve adherence, approximately 27% of participants said they had felt discouraged from taking ART. The question of whether participants had ever been discouraged from using ARVs was designed initially to explore the local world of health-seeking practices, especially with respect to social pressures to use traditional healing for presumptive or actual HIV infection. Participant responses, however, pointed up a myriad of non-mutually exclusive ways that ART discouragement was perpetrated and experienced, and the ways that PLWHA resisted such pressures. Three such ART discouragement processes are described here to illustrate the complexity of social processes that bear on adherence and the ways in which care supporters intervene in these processes.

#### Generalized denial of HIV and AIDS

Demonstrating care supporters’ broader influence over perceptions of HIV and ART, a 32-year-old woman said there were men in her community who discouraged her from taking ARVs, insisting that there is no such thing as HIV and AIDS. “You are being influenced by these caregivers,” they told the woman, “so you must stop taking the tablets.” Prior to her care supporter's intervention, the woman had feared joining others at meals, and on some days she even denied her HIV-positive status. With the care supporter's encouragement, she re-joined her social world and accepted her HIV status, responding to these men's disapprobation: “No, I will not discontinue. I will continue [taking my medications].” She believed in ARVs “because I saw that I was almost dead.”

HIV disclosure emerged from participant reports not just as a complex psychosocial process punctuated by instances of telling different people at different times, but as a means of breaking down others’ denial of HIV and encouraging loved ones to seek ARV treatment. A 60-year-old man was motivated to disclose in order to save his sick brother, hoping that by sharing his own HIV status and invoking his improved health as evidence of ARV effectiveness, his brother would seek testing and treatment. He had told his brother, “Look here, I'm HIV positive, and I've started these ARVs. I was just like you, so now you go and have the HIV test and get these tablets.” The brother reacted by threatening the participant with violence and denying that he himself might have HIV: “He wanted to kill me, because I tried to explain everything to him about the CD4 count. He said, ‘Eh, there's nothing like that’.” Eventually the brother capitulated. On ARVs, the participant said, his brother is “sharp” and thankful that he intervened. Moreover, his brother now “preaches the gospel [of testing and treatment] to other people, that they must go for the HIV test or else they will end up very sick.”

#### ARVs and the association with being HIV positive

The social friction that some participants described was attributable to the association of ART with HIV infection. Therefore, questions regarding feeling discouraged from taking ART elicited accounts of the role that ART played in constituting an HIV-positive identity. This link was evident in the report of a 32-year-old woman who, with the help of her care supporter, decided to tell her sister of her HIV status in the hope that she would seek testing and treatment. Yet self-disclosure ran the risk of further complicating what others believed about HIV and AIDS and ART, especially if an HIV-positive person appeared well as a result of treatment:

The care supporter helped me to tell my family, because my sister was also sick. I was afraid to tell her, because I thought she would say that I am laughing at her, or bluffing. So the care supporter advised me to make an example with my life. But my sister couldn't accept the [HIV] positive life, so she passed on [died].

Despite her sister's death, the woman's family refused to believe that the participant was HIV positive.

My family didn't believe I was being helped by the ARVs to get well. They said I was just telling stories. Since my sister died, [though], they try to believe me …. To my husband's family, I decided on my own to tell them I am living positively. Even they don't believe I have HIV [apparently because participant appeared well]. My mother-in-law reminds me when to take the ARVs, but doesn't believe I have HIV.

Asked how her family's unwillingness to believe she was HIV positive made her feel, she said, “It hurts me a lot, because they're busy dying left and right; because they do not believe what I am saying. So I pray one day that they may accept that I am HIV positive.” To accept that the participant was HIV positive required, at the same time, that they believe in the potential effectiveness of ARVs. Unfortunately, to believe in ARVs was to concede the harsh and frightening reality of HIV and AIDS, which some individuals reportedly were unwilling to do.

A second participant similarly described the simultaneous denigration of being HIV positive and taking ARVs perpetrated by his colleagues at work (as a taxi driver). They called him stupid, he said, for taking the ARVs, claiming that if they were HIV positive, they would not need to rely on the tablets as he does. “They think they are clever,” said the participant. “They are stupid.” Asked how he reacts, he said, “I just keep quiet, because I know what I'm doing for my life.”

A third participant reported being disparaged and discouraged from taking ARVs by members of her community but of pushing back vocally. “Even if I take the ARVs,” she told them, “I am very fortunate because I know my [HIV] status. What about you? It's highly possible that you have only five CD4 count.” That she invoked self-knowledge of her CD4 count as a sign not of inferiority but as a source of defiant pride suggested not just a heightened degree of HIV and AIDS literacy but the deployment of that literacy in the service of combatting stigma.

Associating an individual's ARV regimen with social transgression was another means by which participants might be discouraged. Some people, explained another participant, felt it was a disgrace to take ARVs in front of other people, for example, at a funeral. Since funerals might run all night, such transgressions might be necessary if adherence was to be achieved. The power of social relations to subvert an individual's efforts to adhere to ART was evident in the case of a 42-year-old widow whose husband had been supportive of her. Unfortunately, her in-laws protested her use of ARVs, refusing to enter her house or eat any food that she prepared, because “you are [taking] ARVs.” The participant said they “hated” that she was on ART.

#### Pressure to use non-biomedical treatment modalities

A third means of discouraging an individual from taking ARVs was to challenge the participant's confidence in the clinical efficacy of ART. A 2008 Swazi government report found that beliefs in the effectiveness of traditional healing for HIV infection threatened ART uptake as well as adherence [42, p. 57]. At least two participants said their peers had told them that ARVs would make them very sick and that they would die as a result. A participant described a family dispute in which her father-in-law insisted they she must not take ARVs, but rather herbal medicines as ARVs would cause her to die. Asked how she responded to such pressure, the participant said she insisted, “No, you are the one who is going to die.” A 58-year-old woman said people had discouraged her from taking ARVs, pressuring her to use a popular product called “Forever Living” instead. A 56-year-old woman, diagnosed HIV positive in 1999, said she did not yet need ARVs, presumably because her CD4 count indicated ART was not yet warranted. She described how friends counselled that even if the clinic advised her to do so that she must not take them. She resisted, telling them, “I won't [say no to starting ARVs], because I was taught how to care for myself. They teach me that if I am supposed to take ARVs, I must take them my whole life.”

A self-declared commitment to ART adherence was a salient feature of participants’ reports, as was the importance of care supporters’ encouragement to do this. Participants described care supporters’ on-going ART education and encouragement, their counsel to avoid traditional herbal remedies, their readiness to respond to HIV-related questions and advice to attend the clinic as among the “most important” roles they played.

## Discussion

Participant reports were calculated and coded as a means of assessing the frequency and describing the social mechanisms of adherence impact achieved by PLWHA with their care supporters. Inductive analysis of open-ended data pertaining to adherence generated 31 sub-categories that were abstracted into seven main categories (Table 2). This number falls within the three and eight main categories characteristic of general inductive studies [43]. From these categories, there emerged two themes that helped to explain how care supporters achieved a positive adherence impact among participants: (i) strategic “talk” between care supporters and their clients that facilitated adherence, the significance of which was embedded in (ii) care supporters’ continuous real-time engagement in clients’ daily lives. The resulting care practices constituted new modes of sociality whereby the care supporter relationship was the CHBC intervention and primary mechanism of adherence impact.

**Table 2:** General inductive analysis – seven main categories

Category title	Category description
Category 1: Stigma	The role of HIV stigma in being HIV positive, and the subsequent impact of stigma reduction by care supporters
Category 2: Impact	Relief/impact: material, social, subjective, physical achieved by care supporters
Category 3: Talk	The importance of dialogue with the care supporter in transforming the meanings of being HIV positive and HIV-related health practices
Category 4: Household relations	Impact of CHBC services on family care practices and thus on PLWHA experiences of “home”
Category 5: Self-care	Cultivating PLWHA self-care, self-efficacy by encouraging real time and on-going HIV healthy practices
Category 6: Care supporter limitations	Frustration with CHBC limitations
Category 7: Religion	Christianity as a distinctive resource for CHBC HIV and AIDS care

### Strategic “talk” as a social mechanism of adherence impact

By reinforcing clinic counselling and creating opportunities for a deeper understanding of one's diagnosis, care supporter–client talk constituted a social practice with ART adherence implications. It is important to distinguish between HIV and AIDS “talk,” which entails dialogue, and HIV and AIDS education or counselling at clinics, which is often episodic, unilateral and constructed as authoritative by virtue of its biomedical basis. Furthermore, clinic services and mass health campaigns are often limited in their capacity to facilitate HIV and AIDS “talk” at the scale or intensity that is needed to reduce household and community stigma and embolden individual HIV healthy practices. Multiplying the number of safe, constructive and relevant conversations around HIV and AIDS was one of the CHBC's key impact mechanisms. A 44-year-old woman in a polygamous marriage said she sought HIV testing after witnessing her husband's second wife fall very ill. Her husband, she explained, refused to test or use a condom, so she abstained from sex with him. Differentiating clinic from home-based care, the woman explained that while the health centre had provided her with “full counselling,” the “care supporters are nearer to us each and everyday. They are close to us. And we are open to speak to the care supporter about things that we are afraid to speak to the nurses about.” Campbell et al.'s study of AIDS stigma in two South African communities [44] similarly demonstrated the importance of safe social spaces, noting “that what people lack is not always information, but rather social spaces in which they feel safe to discuss HIV and AIDS” (p. 409).

CHBC facilitation of HIV disclosure, and its potential impact for strengthening HIV prevention through integrated health services, has been little described in the literature [45, p. 266]. Similarly, CHBC facilitation of HIV disclosure and its impact on strengthening ART adherence remains underexplored. Speaking against collective misinformation and HIV stigma, care supporters’ real time and on-going conversations – talk – emerged from participant reports as powerful instruments of individual wellbeing and, potentially, household and community health as well. The notion of “talk” as a dynamic variable that could ripple from the primary client-care supporter relationship to other household members was noteworthy, since substantial CHBC impact derived from on-going conversations between the care supporter and individual client as well as, in some instances, family members. It was here, in the home of the client, that different aspects of being HIV positive could be safely – safe from judgement and inaccurate information – discussed.

A 43-year-old participant said she had felt unwell and that she had visited a number of clinics but had experienced no improvement. Illustrating the significance of HIV and AIDS talk as a social process that facilitates HIV healthy practices over time, the woman described how a care supporter encouraged her to request an HIV test. Having done so, she learned that she was HIV positive. Once her CD4 count was checked, she returned to her care supporter to discuss next steps. The care supporter asked the woman about her own thoughts, to which the participant replied, “There is no alternative. I accept it. I am going to take the ARVs, so I started.” Discriminated against by her family, in part because of their negative views of ARVs, the care supporter continued to play an indispensable role in the woman's survival, acting as both mother and pastor to ease her pain. When her 25-year-old daughter died, recalled the participant, neither family members nor fellow church parishioners nor her pastor attended the funeral. It was her care supporter, flanked by other care supporters, who were present.

### The CHBC relationship as ART adherence intervention

Published data have suggested that CHBC may substantially strengthen adherence and responses to ART in rural, resource-limited settings, especially where transportation to a health centre presents an obstacle to adherence [46, 47, p. 8] as was the case in this study. Supporting the ethnographic findings of Ware et al. [48] of “economic obstacles and the strength of social relationships as principal mediators of sustained adherence in sub-Saharan Africa” (p. 45), participants in this study described how changes in HIV healthy practices, including adherence, were often embedded in the new social relationships and material assistance afforded by care supporters. Given the scale at which the SHBC operates, care supporter relationships engendered a broader social process of mitigating peer pressure against using ART that came from clients’ friends, family members, colleagues and others. The negative impact of perceived stigma on adherence in particular suggested that care supporters constituted a vital source of social capital, one which participants did not have to initiate or coordinate. The proactive dynamic of CHBC interventions through relationship formation was critical since HIV stigma, combined with sickness, can make it impossible for an individual compromised by HIV and poverty to mobilize social networks.

Care supporters’ targeted concern with assisting clients to adhere was often integral to relationship building. This meant that care supporters immersed themselves in the tragedies and tribulations their clients faced, and which, importantly, care supporters themselves may suffer. A 30-year-old participant described how her care supporters helped integrate the diagnostic reality of her HIV-positive status into her understanding of what it means to have HIV and AIDS and thereby adhere to treatment: “I don't understand things about the sickness,” she explained. “At times, I may think I'm not HIV positive and may want to stop the ARVs, but [the care supporters] answer my questions.”

SHBC exercised its impact in part by helping participants to cultivate self-efficacy, which has been described as “the degree to which a person feels that he or she has control over important aspects of his or her life” [49, p. 72–73]. The ritualized and real-time nature of care supporter involvement in clients’ lives helped to develop self-efficacy skills in ways sporadic clinic visits are not designed to do. In contrast, self-efficacy in the context of CHBC was mediated through the formation of new modes of social relationships. These were twofold: dyadic between care supporters and clients, and sometimes as collectivities that included clients, family members, and care supporters (some of who might also be HIV positive). Escott et al. [50] similarly identified the social aspect of treatment support in a community-based TB DOTS Programme in Swaziland, noting “the role of the treatment supporter is wider than being just a DOT provider—more than just observation of treatment” (p. 1707). Findings from this study similarly showed adherence to be more than just a regimen, but rather a social practice embedded in client and care supporter relationships.

Participants described intense social pressures they faced to desist from their ART regimen, which further highlighted the importance of care supporters to reduce social barriers to uptake and embolden individuals’ decisions to adhere to their prescribed ART regimens. Mills et al. [13] have pointed up the importance of “understanding culturally specific barriers to adherence” (p. 688). Yet, rather as “barriers,” it may be productive to conceptualize discouragement and resistance to ARVs as social processes that reflected the diverse and contested views not only of HIV and AIDS but of ART as well. For example, in a five-country study of HIV stigma and ART that included Swaziland, Makoae et al. [51] found that though HIV stigma declined over time, being on ART sometimes entailed stigma, possibly because ART-related health practices cued others to an HIV-positive status. Because ARV discouragement overlapped with HIV stigma and persistent HIV and AIDS denial, participant reports pointedly illustrated the cultural complexity that care supporters were often uniquely well equipped to navigate. Many conventional HIV and AIDS programmes have yet to address these serious and complex obstacles, for which more studies are needed that are specific to cultural settings. Critical among these is addressing how HIV stigma plays out through multiple modalities [52] to complicate ART adherence, often by pre-empting a strong HIV-positive identity. Participants’ insights on the roles that care supporters played in supporting both adherence and the formation of a strong HIV-positive identity demonstrated the situated importance of CHBC to developing “health enabling community contexts” [53, p. 508]. These overlapping processes were evident in the case of the widow who said her in-laws would enter her home only if she stopped taking her ARVs. Cessation of medication would, in their minds, presumably erase the fact that she was HIV positive. A 48-year-old woman said her neighbours discouraged her from taking the ARVs because it ran the risk of publicizing her positive HIV diagnosis. The woman responded that she would not stop, because “it's my life,” a discourse of self-ownership echoed by other participants that care supporters strengthened through on-going education and encouragement.

## Conclusions and recommendations

The configurations of CHBC success remain little understood, a knowledge caesura explained in part by public health research frameworks that are limited in their ability to capture the social experience of disease [54, p. 6]. In this article, qualitative health research demonstrated that cultivated social relationships between care supporters and PLWHA were integral to individual HIV-related health practices. Grasping the centrality of these relationships to ART adherence is important because ART has rendered HIV and AIDS a chronic condition in many parts of the world, so long as availability, uptake and adherence are sustained.

It is important to recognize that coverage rates do not equate with chronicity. Where achieving scale has been a key objective of ART rollout policies, the complexities of long-term treatment for increasing numbers of people must be addressed [55, p. 34]. In addition to entrenched structural obstacles, such as lack of clinic transport, participants described the challenges of implementing clinic guidelines upon return home often as a result of food insufficiencies and HIV stigma, each of which can undermine attempts at prevention and treatment among PLWHA [56–58]. In these settings, care supporters were pivotal in their ability to mitigate material scarcity (albeit to a limited degree), counsel side effects management and provide on-going encouragement to persevere under the duress of daily life. As evidenced by thoughts regarding suicide and reports of ART discouragement, this last psychosocial feature of care supporter impact should not be downplayed.

Above all, data from this study made salient the fact that care supporters play extraordinarily powerful and influential roles in PLWHA and their families’ lives. As a consequence, it is imperative that care supporter training be continually monitored and updated regarding new treatment guidelines and other clinical as well as psychosocial insights that bear on PLWHA wellbeing. Care supporters themselves also require appropriately tailored and comprehensive support. Enhanced adherence among CHBC clients may contribute to self-efficacy, which in turn holds out the promise of reducing care supporter burdens. Knowledge of what works to achieve self-efficacy, from PLWHA perspectives, could help to maximize CHBC-limited resources and manage care supporter workloads. Defining and achieving self-efficacy in context is therefore essential for reducing the burden of care that devolves onto community-based health workers through governmental and multi-agency plans throughout Africa to decentralize health services.

Identifying the most meaningful and effective care practices from PLWHA perspectives has been a central goal of this article. Findings are especially relevant to innovative national and global health programming that foresees the possibility that HIV and AIDS, properly managed, can transition from a terminal to a chronic disease in resource-limited settings [59, p. 3]. Research and programming should investigate and incorporate the experiences both of clients and care supporters across CHBC models to identify the configurations best suited to programme goals and community needs. This is especially important in Swaziland, where the National Strategic Framework 2009–2014 has explicitly positioned CHBC as an integral feature of the health services infrastructure that should “alleviate the burden of care at health facilities” [29, p. 58]. The authors recommend the following domains as productive areas of research and policy formulation.

1. Identify and develop effective linkages between CHBC and formal (public health-based) as well as informal (non-public health based) health sectors, including indigenous healers, pharmacists, chemists, herbalists, and prayer healers. Doing so could help to dispel the confusion and misinformation that fuels HIV denialism and discourages ARV adherence.
2. Undertake comparative evaluations of settings where CHBC operates and where none is extant, in order to assess (a) types of CHBC and (b) CHBC practices perceived to be impactful by PLWHA and household members.
3. Conduct focused and comparative studies to determine the significance of stipends and other training/support mechanisms on care supporter wellbeing and retention.
4. Investigate how, in the context of HIV and AIDS chronicity, assistive roles of family, community and clinic are defined and understood to relate to one another.
5. Develop evaluation studies that determine whether there are CHBC ripple effects from clients to household members with respect to HIV-healthy practices, for example, prevention, HIV and TB testing, and treatment uptake/adherence; this would further determine the real impact that CHBC has on an individuals, households, and communities.

These recommendations are aligned with the Joint United Nations Programme on HIV and AIDS (UNAIDS) UNAIDS Treatment 2.0 goal of developing effective HIV and AIDS care delivery systems in order to narrow the treatment gap and strengthen HIV prevention [60, p. 2]. In particular, the authors conclude that a deeper knowledge of PLWHA experiences of CHBC are critical variables in successfully implementing the UNAIDS Treatment 2.0 agenda [61], notably Pillar 4 (increasing HIV testing uptake and care linkages) and Pillar 5 (strengthening community mobilization).

## Authors' details

RR is a medical anthropologist who has investigated socio-cultural aspects of HIV and AIDS in Swaziland since 2005. She is currently an associate professor in the Department of Sociology and Anthropology at Baruch College, The City University of New York.

AW is the Director of the Health Economics and HIV and AIDS Research Division at the University of KwaZulu-Natal. The author of numerous books and articles on HIV and AIDS, he has close links to Swaziland and has been researching HIV and AIDS since 1987. His main research interest is the economic and development impact of HIV and AIDS.
